# Spotlighting SHAPERS: sex hormones associated with psychological and endocrine roles

**DOI:** 10.1038/s41386-024-01819-0

**Published:** 2024-02-16

**Authors:** Nicole Petersen

**Affiliations:** Department of Psychiatry and Biobehavioral Sciences, David Geffen School of Medicine, UCLA, Los Angeles, CA USA

**Keywords:** Neuroscience, Translational research

Imagine that your laboratory discovers a signaling molecule in the brain. This molecule can increase and decrease cortical and subcortical volumes and change brain-wide functional connectome topology. It seems to be related somehow to *most* psychiatric and neurological disorders (epilepsy, stroke, and depressive disorders, to name just a few), conferring risk in some cases and resilience in others. However, you have no information about how this signaling molecule behaves in a living human brain, because in vivo molecular imaging has never been performed [1]. The only information about the distribution of the receptors comes from a handful of postmortem brains. This is the current status of research into the neuroactive steroid 17β-estradiol.

Estradiol is not uniquely mysterious among the neurosteroids. Other estrogens, androgens, progestogens, and their neuroactive metabolites, such as allopregnanolone, remain similarly under-investigated. Here, we approach a nomenclature problem: “Neurosteroids” encompasses all of these molecules, but also includes corticosteroids. “Sex steroids” includes only these reproductive molecules, but also includes reproductive signaling hormones that appear to *only* be involved in reproductive functions, such as follicle-stimulating hormone (However, it seems possible, if not likely, that psychological, non-reproductive effects of all sex steroid hormones may eventually be discovered, obviating the need for this designation.). The goal of this commentary is to focus exclusively on Sex Hormones Associated with Psychological and Endocrine Roles. For this piece I will refer to these as SHAPERs. I invite other investigators to adopt this nomenclature to describe sex steroid hormones that influence psychological domains whenever it is helpful to your work.

Although basic and crucial information about SHAPERs remains unknown, important clinical, translational, and basic science discoveries have recently taken place. Last year, psychiatry witnessed a historic breakthrough when Zurzuvae® (zuranolone) became the first FDA-approved oral treatment for postpartum depression (A previous formulation of this compound, Zulresso® (brexanolone), was FDA approved in 2019, but was largely inaccessible to patients because it was administered as a 60-hour continuous intravenous infusion that could only be given in designated Risk Evaluation and Mitigation Strategies (REMS) sites.). The medication, which is structurally similar to the allopregnanolone molecule and, like allopregnanolone, also acts as a positive allosteric modulator at the GABA_A_ receptor [2], acts rapidly, outperforming placebo effects on depression symptoms within 3 days [3]. This marks the first time in history that individuals with postpartum depression will have accessible, evidence-based, rapid-acting treatment.

Why zuranolone is an effective treatment for postpartum depression remains an area requiring further study. The mechanisms underlying this condition and other adverse responses to hormonal changes are largely unknown. Transcriptomics suggest innate differences in the cellular response to estrogen and progesterone in individuals with vs. those without postpartum depression [4]. Controlled studies of hormone suppression followed by add-back can unmask and reproduce severe negative mood symptoms in some individuals with a history of postpartum depression [5]. Conversely, in a transdiagnostic sample of individuals with suicidal ideation (but no hormone-specific diagnosis) a randomized, controlled, double-blind crossover experiment found that supplemental estrogen and progesterone buffered against the exacerbation of suicidal ideation and other mood symptoms otherwise observed during the perimenstrual period under placebo [6], an effect that appears to be driven by estrogen [7]. These and other careful, rigorous studies show definitively that SHAPERs play a causal role in the etiology of some serious and even life-threatening mood problems, but mechanistic studies are needed to find novel treatment targets and to understand why individuals vary so dramatically in their response to SHAPERs.

Neuroimaging studies have revealed that SHAPERs appear to have substantial influences on both the morphology and connectivity of some – but not all – brain structures. Preclinical work had long since established that SHAPERs, specifically estradiol and progesterone, influence the morphology of medial temporal lobe structures [7] but only recently were these efforts robustly translated by human neuroimaging. Daily imaging of a single research participant revealed significant effects of progesterone on hippocampal subfield volumes [8], which was extended by an ultra-high-field study of 27 densely-sampled individuals. This investigation showed that in humans, 17β-estradiol is positively associated with volume in the parahippocampal cortex, progesterone is positively associated with volume in the perirhinal cortex and subiculum, and the two hormones interact to shape CA1 volume [8]. Both studies reported no relationship between the SHAPERs evaluated and size of the precentral gyrus, indicating that SHAPERs do not produce brain-wide effects – but it remains unknown which brain regions (other than the medial temporal lobe) are also sensitive to the shaping effects of SHAPERs. Similar studies that are designed and powered to address this gap in knowledge are needed.

Just as SHAPERs guide fluctuations in brain structure, they also appear to direct functional connectome topology. Spectral dynamic causal modeling of a large (*N* = 60, within-subjects) and rigorously-characterized group of people revealed elaborate fluctuations both within and between the default mode, salience, and executive control networks during three distinct hormonal windows [9]. These changes are so numerous that they defy simple summarization. A similarly complex pattern of results has emerged from multiple functional connectivity studies of the same densely-sampled individual referenced previously [10–15]. Together, these studies suggest that at least in individuals who experience a menstrual cycle, the connectome is better characterized by cyclical SHAPER-driven network changes than by network stability.

Evidently, SHAPERs produce important effects on brain structure and function that influence human behavior and mental health outcomes. Attention to these important neuromodulators may yield new treatments, as it did in the case of zuranolone, but will require rigorous study using diverse and well-powered datasets. Consortia efforts have begun to form in pursuit of this goal, such as the Ann S. Bowers Women’s Brain Health Initiative and the ENIGMA Neuroendocrinology working group (Readers who are interested in joining this initiative can contact the corresponding author.).

Rather than relegating SHAPER studies to women’s health funding opportunities (men have SHAPERs too!) or neuroendocrinology-specific societies, successfully closing the gaps in knowledge described above will be aided by the enthusiasm of professional organizations, such as the American College of Neuropsychopharmacology and Society of Biological Psychiatry, as well as the financial endorsement of such studies by funding agencies. Together, we can build on the remarkable successes of the recent investigations described here (and many more deserving investigations left out of this short commentary) to untangle these complex problems and discover new, evidence-based treatments for the patients who need them.
